# Risk over time and risk factors of intraoperative respiratory events: a historical cohort study of 14,153 children

**DOI:** 10.1186/1471-2253-14-13

**Published:** 2014-03-05

**Authors:** Maliwan Oofuvong, Alan Frederick Geater, Virasakdi Chongsuvivatwong, Ngamjit Pattaravit, Kanjana Nuanjun

**Affiliations:** 1 Department of Anesthesiology, Faculty of Medicine, Prince of Songkla University, Songkhla 90110, Thailand; 2 Epidemiology Unit, Faculty of Medicine, Prince of Songkla University, Songkhla 90110, Thailand

**Keywords:** Risk over time, Intraoperative period, Respiratory events, Pediatric anesthesia, Tertiary care hospital

## Abstract

**Background:**

The variation in the rate of intraoperative respiratory events (IRE) over time under anesthesia and the influence of anesthesia-related factors have not yet been described. The objectives of this study were to describe the risk over time and the risk factors for IRE in children at a tertiary care hospital in southern Thailand.

**Methods:**

The surveillance anesthetic database and chart review of IRE of 14,153 children who received surgery at Songklanagarind Hospital during January 2005 to December 2011 were used to obtain demographic, surgical and anesthesia-related data. Incidence density of IRE per person-time was determined by a Poisson modelling. Risk of IRE over time was displayed using Kaplan Meier survival and Nelson-Aalen curves. Multivariate Cox regression was employed to identify independent predictors for IRE. Adjusted hazard ratios (HR) and their 95% confidence intervals (CI) were obtained from the final Cox model.

**Results:**

Overall, IRE occurred in 315 out of 14,153 children. The number (%) of desaturation, wheezing or bronchospasm, laryngospasm, reintubation and upper airway obstruction were 235 (54%), 101 (23%), 75 (17%), 21 (5%) and 4 (1%) out of 315 IRE, respectively. The incidence density per 100,000 person-minutes of IRE at the induction period (61.3) was higher than that in the maintenance (13.7) and emergence periods (16.5) (p < 0.001). The risk of desaturation, wheezing and laryngospasm was highest during the first 15, 20 and 30 minutes of anesthesia, respectively. After adjusting for age, history of respiratory disease and American Society of Anesthesiologist (ASA) classification, anesthesia-related risk factors for laryngospasm were assisted ventilation via facemask (HR: 18.1, 95% CI: 6.4-51.4) or laryngeal mask airway (HR: 12.5, 95% CI: 4.6-33.9) compared to controlled ventilation via endotracheal tube (p < 0.001), and desflurane (HR: 11.0, 95% CI: 5.1-23.9) compared to sevoflurane anesthesia (p < 0.001).

**Conclusions:**

IRE risk was highest in the induction and early maintenance period. Assisted ventilation via facemask or LMA and desflurane anesthesia were anesthesia-related risk factors for laryngospasm. Therefore, anesthesiologists should pay more attention during the induction and early maintenance period especially when certain airway devices incorporated with assisted ventilation or desflurane are used.

## Background

Despite the low rate of mortality associated with cardiac arrest in anesthetized children over the past few decades [1], as many as 27% of perioperative cardiac arrests were caused by respiratory events in the Pediatric Perioperative Cardiac Arrest registry [2]. Tait et al. [3] reported an incidence of 1.7% for respiratory events during the intraoperative period in a retrospective study in children with upper respiratory tract infection (URI). A Thai Anesthesia Incidents (THAI) prospective study also reported a similarly low incidence (1.5%) of perioperative respiratory events (PRE) in 25,098 Thai children [4]. In contrast, four recent prospective studies have reported incidences of 15% for PRE in general child populations or as high as 28% in high risk children such as those with URI or obesity [5-8]. However, the separate incidence of intraoperative respiratory events (IRE) was mentioned in only one of those studies [6]. Known risk factors for PRE include history of upper respiratory tract infection, age less than 6 years, obesity, airway surgery, and absence of a specialized pediatric anesthesiologist during the operation [5-8]. However, descriptive profiles concerning changes in the rate of IRE over time in children as well as incidence density of IRE have never been described. Parents often ask whether the anesthetic risk increases with time under anesthesia. The period of anesthesia that anesthesiologists should be most concerned about is still debatable. Moreover, the predictive ability of some characteristics of anesthesia, such as anesthetic technique, related to the change of rate of IRE over time, has also never been investigated. Therefore, the objectives of this study were to describe the risk profile of IRE over time and identify the risk factors for IRE related to patient, surgical and anesthesia profiles.

## Methods

A historical cohort study was conducted at Songklanagarind Hospital, an 853-bed tertiary care hospital in southern Thailand, after approval by the Ethics Committee, Prince of Songkla University [EC 552100813]. Written informed consent to participate in the study was waived. The surveillance anesthetic database under quality assurance by nurse anesthetists was used to identify IRE in children aged less than 15 years undergoing surgical procedures as well as digital imaging or other interventions under general anesthesia (GA) with or without regional anesthesia or peripheral nerve block between the period of January 2005 and December 2011. All identified IRE were confirmed by chart review of either paper-based or electronic anesthetic records which were available since 2005. The anesthetic records are divided into 2 sections; intraoperative section and post-anesthetic care unit (PACU) section where vital signs are routinely recorded every 5 minutes thoughout the anesthetic procedure. The timing of any respiratory events or rescue medication is recorded in the event section in the anesthetic record.

### Participants

All procedures including regional anesthesia or peripheral nerve block were done by a certified anesthesiologist with at least one year’s experience. Patients were excluded if they were classified as American Society of Anesthesiologists (ASA) physical status 4 or 5, had a preoperative arterial oxygen saturation at room air < 95%, were already endotracheally intubated and/or mechanically ventilated prior to surgery or had congenital heart disease with cyanosis. These criteria meant that existing hypoxemic children or children who previously had severe respiratory or circulatory problems were excluded.

### Anesthesia practice

In routine practice in the hospital during the period of study, the choices of anesthetic technique, airway devices and anesthetic agents were at the discretion of the attending anesthesiologists. Attending anesthesiologists supervised all anesthesia residents and nurse anesthetists for all anesthetized children from the start of anesthesia until discharge from the PACU. There was a total of 28 attending anesthesiologists throughout the 7 year study period. The average number of attending anesthesiologists each year was 17; 2 were certified pediatric anesthesiologists, 8 had experience in pediatric anesthesia > 5 years and 7 had experience < 5 years. The induction technique for GA was either inhalation using sevoflurane or intravenous injection. The maintenance of anesthesia was either by spontaneous breathing with 100% oxygen using intravenous anesthesia, or by facemask or laryngeal mask airway (LMA) using inhalation anesthetic agent with N_2_O-O_2_ or Air-O_2_, or by endotracheal tube (ETT) using neuromuscular blocking agent (NMBA).

#### Standard operating procedures

The mode of ventilation was either by manually assisted ventilation or controlled ventilation by anesthetic machine ventilator. For manually assisted ventilation via facemask or LMA, tidal volume was adjusted at 7-10 ml/kg by anesthesia personnel and synchronised with children’s spontaneous breathing. Assisted ventilation by anesthetic machine ventilator was not applied if children could breathe spontaneously. For ETT under controlled mechanical ventilation, the anesthetic machine ventilator was set at the same tidal volume. Positive end-expiratory pressure was not routinely applied during controlled ventilation in children except in prolonged (more than 3 hours) anesthesia.

One hundred percent oxygen with high flow (5 liters per minute) was applied at least one minute before intubation or LMA insertion and immediately after extubation. Basic intraoperative monitoring including pulse oximetry and end tidal carbon dioxide were used during the period of anesthesia. After the operation, children were sent either to the PACU or to the pediatric intensive care unit (PICU) depending on the decision of the surgeon or anesthesiologist. One hundred percent oxygen was not usually administered while relocating children to the PACU unless persistent desaturation (arterial oxygen saturation < 95% with 100% oxygen more than one minute) occurred before transportation.

### Outcome of interest and variables

Laryngospasm, wheezing or bronchospasm, hypoxemia or desaturation (oxygen saturation < 95% for more than 10 seconds [9]), upper airway obstruction (UAO; soft tissue obstruction or secretion obstruction after the airway device was removed) and re-intubation were chosen as significant events constituting IRE based on their seriousness which can lead to severe hypoxemia and cardiac arrest in children. The categories of IRE were not mutually exclusive. Thus, laryngospasm, bronchospasm and UAO might have desaturation as a result. Time to first IRE was the outcome of interest, assessed by the researcher by performing chart review of all IRE anesthetic records during the data management period. Cause of desaturation was also assessed.

Variables collected were categorised into patient-related risk factors, surgical-related risk factors and anesthesia-related risk factors. Patient-related risk factors were age, sex, weight (kg), height (cm), body mass index (kg/m^2^), recent upper respiratory tract infection (URI), pulmonary disease such as asthma or pneumonia, allergic rhinitis, food or drug allergy, non-cyanotic heart disease, anemia (hematocrit < 39% in male and < 36% in female). Surgical-related risk factors were type of surgery (elective/emergency), type of patient (inpatient/outpatient), position (supine, lateral, lithotomy, prone) and site of operation (superficial; chest wall, abdominal wall and minor surgery, bronchoscopy or laryngoscopy, ear-nose-throat, eye, intrathoracic, intraabdominal, spinal or extremity, remote; radiology services and cardiac catheterization). Anesthesia-related risk factors were ASA classification, choice of anesthesia (GA, GA with regional anesthesia, GA with peripheral nerve block), premedication agent (none, chloral hydrate, diazepam, others), induction anesthetic agent (propofol, thiopental, sevoflurane, others), intubating agent (non-depolarizing muscle relaxant, succinylcholine, sevoflurane), narcotic (fentanyl, morphine, others, none), airway device with mode of ventilation (ETT-controlled ventilation, jet ventilation-controlled ventilation, facemask-assisted ventilation, LMA-assisted ventilation), inhalation agent (sevoflurane, isoflurane, halothane, desflurane), gas mixed with oxygen (Air-O_2_, N_2_O-O_2_, 100% O_2_), use of a NMBA and experience of key attending anesthesiologist (< 5 years, > 5 years, certified pediatric anesthesiologist).

Missing data from the anesthetic database were completed by hospital information system or the electronic anesthetic record by the researcher. Height was missing from 1.4% of records and was replaced with the mean height of the same age and sex.

#### Definitions of periods of anesthesia

The period of anesthesia of the 14,153 records and the timings of first IRE was divided into 3 periods of anesthesia; induction, maintenance and emergence periods. The induction period was defined as the time between starting of anesthesia and the starting of incision. The maintenance period was defined as the time between starting of incision and finishing the operation. The emergence period was defined as the time between finishing the operation and the airway device being removed. The 3 periods of anesthesia were defined using variable cut points of time based on the total duration of anesthesia adjustable by airway device used. For the total duration of anesthesia more than 30 minutes, the induction and emergence periods were 15 minutes after starting anesthesia and 15 minutes before ending of anesthesia, respectively, because most children received ETT intubation or LMA insertion. For children in which the total duration of anesthesia was less than 30 minutes, the induction and emergence periods were 5 minutes after starting anesthesia and 10 minutes before ending anesthesia, respectively, because most children received facemask ventilation with spontaneous breathing. The maintenance period was calculated based on subtraction from the total period of anesthesia of the combined duration of induction and emergence periods.

### Statistical analysis

Descriptive statistics were used to summarize characteristics of the patients. The incidence density of any IRE per person-time (person-minutes) based on period of anesthesia and duration of each period was calculated by fitting a Poisson regression model. Significant differences of incidence density of any IRE among the periods of anesthesia were determined if the p-value from the likelihood ratio test was less than 0.05.

Time to first occurrence of IRE was calculated from start of GA until IRE or, if no IRE occurred, patients were considered as censored at the end of GA. Risk of occurrence of IRE over time was displayed using Kaplan Meier and Nelson-Aalen curves [10]. Log rank tests were initially employed to assess the differences in time-to-event across the subgroups of potential predictor variables. Cox regression models were fit to identify independent predictors of IRE based on the timings of first events of any IRE, laryngospasm, desaturation, wheezing or bronchospasm, or UAO in combination with reintubation. After running the multivariate Cox regression model, variables which violated the proportional hazard assumption were considered to have time-varying effects and explored further to determine if their effects differed among the 3 periods (induction, maintenance and emergence). This was done by creating 3 partial proxy variables for each variable violating the proportional hazard assumption and fitting them in place of the original variable. Adjusted hazard ratios and their 95% confidence intervals (CI) were obtained from the final Cox model. In multivariate modeling techniques, an association with the outcome was considered statistically significant if the likelihood ratio p-value was less than 0.05.

### Sample size considerations

Adequacy of sample size for identifying the risk factors for IRE was based on a preliminary examination of a subset of the children. The IRE-free Kaplan-Meier profile of 2,109 children undergoing GA in 2010, among whom 44 events were recorded, revealed an event-free probability of approximately 0.975 corresponding to a GA duration of 2.5 hours. With a total sample of approximately 14,000 children and a prevalence of exposure to a risk factor between 5% and 50%, differences in 1-hour risk of IRE as small as 2% to 1% (depending on prevalence) should be detectable as significant at an alpha of 0.05 and a power of 80%. We therefore believed that a sample size of 14,000 would be adequate.

## Results

A total of 16,316 children undergoing GA from 2005 to 2011 were identified under the period of data management between May 2012 and December 2012. We excluded 1,552 cyanotic heart disease and/or required endotracheally intubated prior to surgery, 507 ASA classification ≥ 4 and 104 preoperative arterial oxygen saturation at room air < 95%. IRE occurred in 315 (2.23%) of the 14,153 study children, the numbers (%) of desaturation, wheezing or bronchospasm, laryngospasm, reintubation and UAO events were 235 (54%), 101(23%), 75 (17%), 21 (5%) and 4 (1%), respectively. The causes of intraoperative desaturation (235 children) were light anesthesia (children were not adequately anesthetized) with endotracheal tube placed (31%), laryngospasm (26%), bronchospasm (22%), prolonged apnea during application of laryngoscopy (12%), accidental extubation (4%), endobronchial intubation (1.4%) and UAO (1.4%). The median duration of anesthesia was 90 minutes (interquatile range (60,140 minutes)). One hundred and nineteen children developed more than 1 IRE. Table 1 shows the number and incidence density of IRE per 100,000 person-minutes among each period of anesthesia. The incidence density of each type of IRE except UAO in combination with reintubation was highest in the induction period of anesthesia (p < 0.001).

**Table 1:** Number and incidence density of intraoperative respiratory events (IRE) per 100,000 person-minutes among 14,153 children

**Period of anesthesia (person-minutes)**	**Induction (200,584)**	**Maintenance (1,156,812)**	**Emergence (206,453)**	**p-value**
**IRE**	**Number (incidence per 100,000 person-minutes)**			
Desaturation	90 (44.9)	124 (10.7)	21 (10.2)	< 0.001
Wheezing	48 (23.9)	41 (3.5)	12 (5.8)	< 0.001
Laryngospasm	27 (13.5)	38 (3.3)	10 (4.8)	< 0.001
UAO & reintubation	1 (0.5)	15 (1.3)	7 (3.4)	0.056
Any	123 (61.3)	158 (13.7)	34 (16.5)	< 0.001

p-value from likelihood ratio test.

UAO: Upper airway obstruction.

### Characteristic profiles of intraoperative desaturation, wheezing, laryngospasm and UAO in combination with reintubation

**Figure 1:**
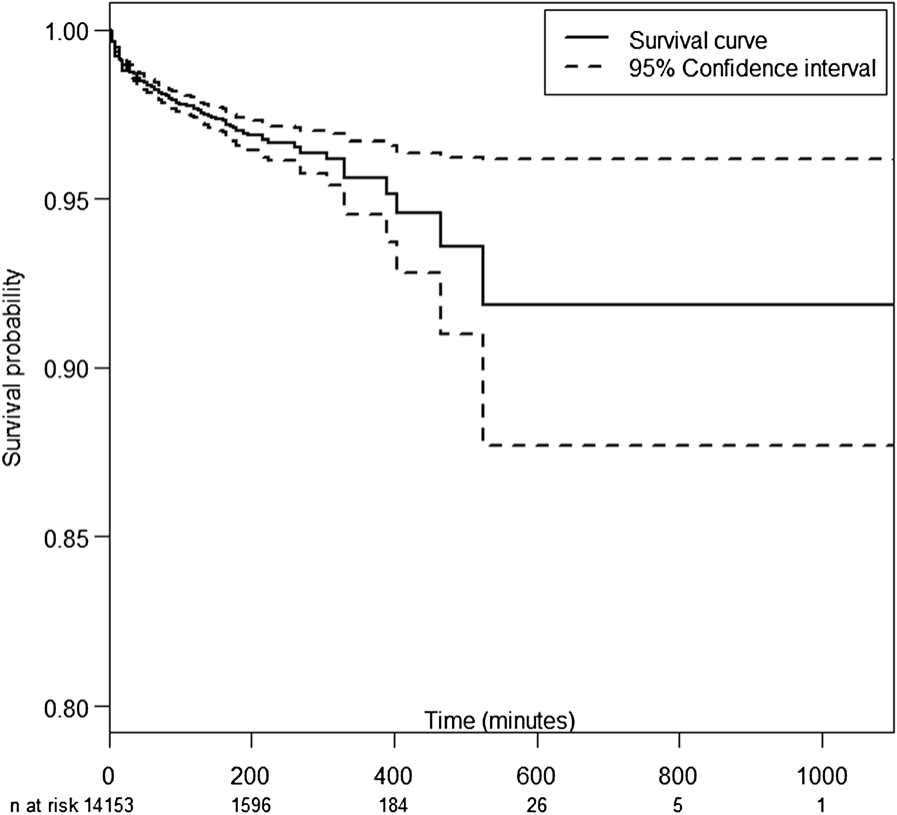
Kaplan Meier survival curve of the first intraoperative respiratory event.

**Figure 2:**
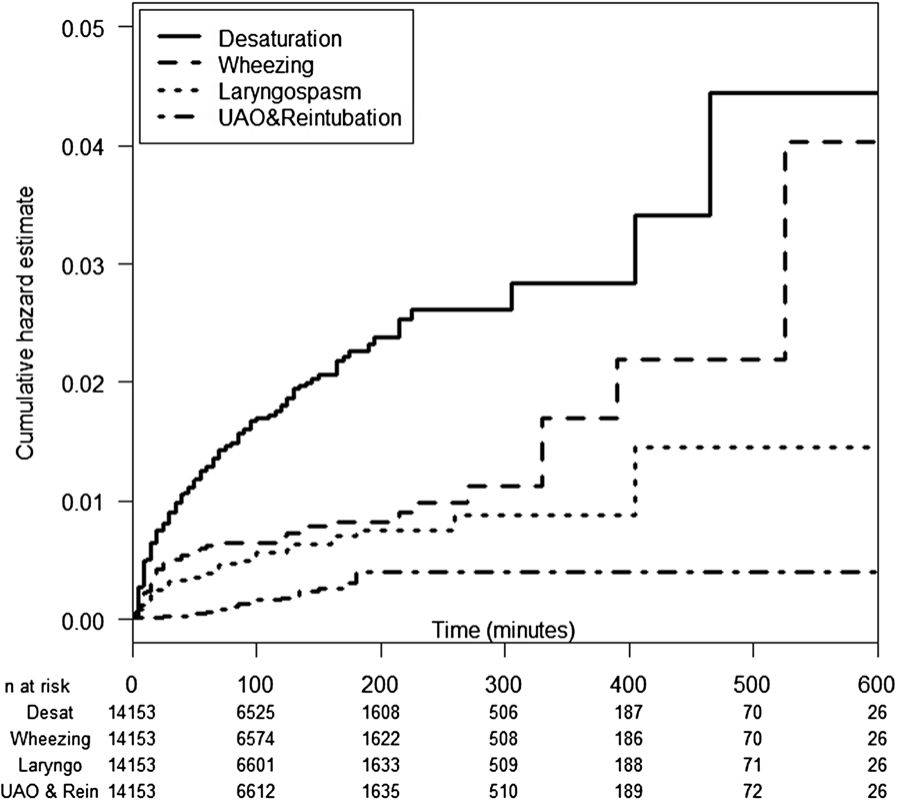
**Nelson-Aalen plot of the first event of different forms of intraoperative respiratory event.** Desat; Desaturation, Laryngo; Laryngospasm, UAO & rein; Upper airway obstruction in combination with reintubation.

Figure 1 shows the IRE-free Kaplan-Meier curve. The probability of being IRE free after 500 minutes was 0.92. IRE was found in all periods of anesthesia including the induction (38%), maintenance (43%) and emergence periods (19%). However, these percentages do not reflect the actual risk of IRE during these periods since the number of subjects at risk in the later periods declines over time. More appropriately, Figure 2 shows the Nelson Aalen plot of cumulative hazard of IRE describing the change in rate of different forms of IRE over time by the slope of the cumulative curve.

The curve for desaturation was steepest during the first 15 minutes, while upward convexity was evident in the first 100 minutes indicating lowering of risks over this period. After 100 minutes, the convexity gradually disappeared suggesting that the risk had stabilized.

Other IRE included wheezing, laryngospasm and UAO and reintubation. Their cumulative hazard profile had different shapes. The profile for wheezing was steepest during the first 20 minutes and stabilized after 1 hour. After 300 minutes, the curve had somewhat of an upward concave shape, suggesting increasing risk after around 5 hours of GA. The curve for laryngospasm was steepest during the first 30 minutes and then stabilized with no laryngospasm occurring after 400 minutes. By contrast, the curve for UAO in combination with reintubation exhibited an upward concave shape, indicating a steadily increasing risk over the first 180 minutes; however, there was no subsequent event after that time.

### Univariate and Cox regression analysis of IRE

The univariate Kaplan Meier analysis for overall IRE included patient-related (8 variables), surgery-related (4 variables) and anesthesia-related (11 variables) risk factors (see Additional file 1). The overall IRE probability (95% CI) of IRE within 60 minutes of anesthesia was 0.017 (0.015, 0.019). Fourteen potential predictors which had log rank p values ≤ 0.2 were included in the initial multivariate Cox regression analysis for IRE. After model refinement, 8 out of the 14 potential predictors remained in the final model (see Additional file 1). Age, history of respiratory disease and ASA classification violated the assumption of proportional hazards and each was therefore used to create 3 partial proxy variables to allow their effects to differ among induction, maintenance and emergence periods and the dataset reorganized into multirecord format. Table 2 shows the results of multivariate Cox regression of IRE incorporating these time-varying effects. The significant risk factors were lateral position, airway surgery, airway device with mode of ventilation, use of succinylcholine as an intubating agent and desflurane anesthesia, whereas age, history of respiratory disease and ASA classification were allowed to have time-varying effects. It should be noted that children whose airway received controlled ventilation via ETT had 2.0- to 2.4-fold lower risk than other groups (p < 0.001). During induction period, an elevated risk was associated with age < 1 years (p = 0.004), having pulmonary disease (p < 0.001) and ASA classification 3 (p < 0.001). In the maintenance period, risk was elevated in patients age ≤ 6 years (p < 0.001) and having URI (p = 0.008), while during the emergence period, a higher risk was associated with age < 1 years (p = 0.048).

**Table 2:** Multivariate Cox regression of IRE based on period of anesthesia (n = 42,055)

**Variable**	**All periods (315)**	**Induction (123)**	**Maintenance (158)**	**Emergence (34)**
	**Adjusted hazard ratio (95% CI) p-value**	**Adjusted hazard ratio (95% CI) p-value**	**Adjusted hazard ratio (95% CI) p-value**	**Adjusted hazard ratio (95% CI) p-value**
Age (ref= > 6)	_	1^a^ 0.004	1^a^ < 0.001	1^a^ 0.048
1-6		1.5 (0.94, 2.3)^a^	2.3 (1.6, 3.4)^b^	2.0 (1.0, 4.1)^a^
< 1		2.5 (1.5, 4.2)^b^	3.0 (1.8, 4.8)^b^	3.1 (1.2, 8.2)^b^
Respiratory disease (ref = no)	_	1^a^ < 0.001	1^a^ 0.008	1^a^ 0.2
URI		1.4 (0.67, 2.9)^a^	2.2 (1.3, 3.7)^b^	2.1 (0.72, 6.3)^a^
Pulmonary disease		3.5 (2.3, 5.5)^b^	1.6 (0.9, 2.8)^a^	2.1 (0.81, 5.7)^a^
Site of operation (ref = superficial)	1^a^ < 0.001	_	_	_
Broncho/laryngoscopy	3.6 (2.0, 6.6)^b^			
Ear-nose-throat	1.6 (0.86, 2.8)^a^			
Eye	1.0 (0.55, 1.8)^a^			
Thoracic	1.1 (0.59, 2.2)^a^			
Abdomen	1.4 (0.83, 2.4)^a^			
Spine & extremity	1.3 (0.74, 2.3)^a^			
Remote*	1.3 (0.48, 2.8)^a^			
Position (ref = supine)	1^a^ 0.009	_	_	_
Lateral	2.0 (1.3, 3.1)^b^			
Lithotomy	0.50 (0.23, 1.1)^a^			
Prone	1.1 (0.51, 2.2)^a b^			
ASA classification (ref = 1)		1^a^ < 0.001	1^a^ 0.83	1^a^ 0.75
2	_	1.7 (0.96, 3.1)^a^	1.0 (0.66,1.5)^a^	1.3 (0.64, 2.7)^a^
3		4.0 (2.1, 7.8)^b^	1.1 (0.66, 2.0)^a^	1.2 (0.38, 3.8)^a^
Airway device (ref = ETT-C)	1^a^ < 0.001			
Jet ventilation-C	2.4 (1.1, 5.1)^b^	_	_	_
Facemask-A	2.4 (1.4, 4.0)^b^			
LMA-A	2.0 (1.2, 3.3)^b^			
Intubating agent (ref = NDMR)	1^a^ < 0.001			
Succinylcholine	1.7 (1.2, 2.2)^b^	_	_	_
Sevoflurane	1.6 (0.9, 2.7)^ab^			
Inhalation agent (ref = sevoflurane)	1^a^ 0.0013			
Isoflurane	0.77 (0.58, 1.0)^a^			
Halothane	0.82 (0.26, 2.6)^a^	_	_	_
Desflurane	3.9 (2.1, 7.2)^b^			

p-value from likelihood ratio test, ASA: American Society of Anesthesiologist; CI: Confidence interval; ETT-C: Endotracheal tube with controlled ventilation; LMA-A: Laryngeal mask airway with assisted ventilation; NDMR: Non-depolarizing muscle relaxant; URI: Upper respiratory tract infection, *remote sites including radiology service and cardiac catheterization, hazard ratios within columns and variables that have no superscript (^a^^b^) in common differ significantly at p < 0.05 (Wald test).

Tables 3, 4, 5, 6 show the risk factors for different forms of IRE. The risk factors for desaturation were similar to those for any IRE. The risk factors for wheezing were having URI or pulmonary disease (p < 0.001) and ASA classification 3 (p = 0.014). The risk factors for laryngospasm were having pulmonary disease (p = 0.02), assisted ventilation via facemask or LMA compared to controlled ventilation by ETT (p < 0.001) and desflurane anesthesia (p < 0.001), whereas age and intubating agent were the factors with time-varying effects. The probability of laryngospasm during the induction period was increased by the use of succinylcholine (p < 0.001), in the maintenance period by 1-6 years of age (p = 0.004) and at the emergence period by ≤ 6 years of age (p = 0.002). The only risk factor for UAO in combination with reintubation was airway surgery, whereas age was an effect-varying factor with probability of UAO in combination with reintubation in the maintenance and emergence periods increased by < 1 years of age (p = 0.01 and p = 0.005, respectively).

**Table 3:** Multivariate Cox regression of desaturation based on period of anesthesia (n = 42,055)

**Variable**	**All periods (235)**	**Induction (90)**	**Maintenance (124)**	**Emergence (21)**
	**Adjusted hazard ratio (95% CI) p-value**	**Adjusted hazard ratio (95% CI) p-value**	**Adjusted hazard ratio (95% CI) p-value**	**Adjusted hazard ratio (95% CI) p-value**
Age (ref= > 6)	1^a^ < 0.001			
1-6	2.3 (1.6, 3.3)^b^	_	_	_
< 1	3.7 (2.5, 5.6)^c^			
Respiratory disease (ref = no)		1^a^ < 0.001	1^a^ 0.16	1^a^ 0.6
URI	_	1.1 (0.45, 2.9)^a^	1.7 (0.89, 3.2)^a^	1.5 (0.34, 6.8)^a^
Pulmonary disease		3.9 (2.4, 6.3)^b^	1.6 (0.84, 2.9)^a^	1.9 (0.54, 6.5)^a^
Site of operation (ref = superficial)	1^a^ < 0.001			
Broncho/laryngoscopy	4.1 (2.1, 8.3)^b^			
Ear-nose-throat	1.7 (0.87, 3.4)^a^			
Eye	1.0 (0.49, 2.1)^a^	_	_	_
Thoracic	1.3 (0.62, 2.8)^a^			
Abdomen	1.3 (0.70, 2.4)^a^			
Spine & extremity	1.2 (0.63, 2.4)^a^			
Remote*	1.7 (0.64, 4.3)^a^			
Position (ref = supine)	1^a^ 0.01			
Lateral	2.0 (1.2, 3.3)^b^	_	_	_
Lithotomy	0.42 (0.15, 1.2)^a^			
Prone	0.77 (0.28, 2.1)^a^			
ASA classification (ref = 1)		1^a^ < 0.001	1^a^ 0.75	1^a^ 0.93
2	_	1.9 (0.92, 3.9)^a^	1.1 (0.66,1.7)^a^	1.1 (0.56, 2.2)^a^
3		4.4 (2.0, 9.6)^b^	1.3 (0.70, 2.3)^a^	0.90 (0.25, 3.1)^a^
Airway device (ref = ETT-C)	1^a^ 0.005			
Jet ventilation-C	2.2 (0.89, 5.3)^a^	_	_	_
Facemask-A	2.5 (1.3, 4.7)^b^			
LMA-A	2.0 (1.1, 3.6)^b^			
Intubating agent (ref = NDMR)	1^a^ < 0.001			
Succinylcholine	2.0 (1.4, 2.8)^b^	_	_	_
Sevoflurane	1.2 (0.62, 2.4)^a^			
Inhalation agent (ref = sevoflurane)	1^a^ < 0.001			
Isoflurane	0.79 (0.57, 1.1)^a^			
Halothane	1.2 (0.40, 3.8)^a^	_	_	_
Desflurane	5.7 (3.1, 10.7)^b^			

p-value from likelihood ratio test, ASA: American Society of Anesthesiologist; CI: Confidence interval; ETT-C: Endotracheal tube with controlled ventilation; LMA-A: Laryngeal mask airway with assisted ventilation; NDMR: Non-depolarizing muscle relaxant; URI: Upper respiratory tract infection, *remote sites including radiology service and cardiac catheterization, hazard ratios within columns and variables that have no superscript (^a^^bc^) in common differ significantly at p < 0.05 (Wald test).

**Table 4:** Multivariate Cox regression of wheezing based on period of anesthesia (n = 42,055)

**Variable**	**All periods (101)**	**Induction (48)**	**Maintenance (41)**	**Emergence (12)**
	**Adjusted hazard ratio (95% CI) p-value**	**Adjusted hazard ratio (95% CI) p-value**	**Adjusted hazard ratio (95% CI) p-value**	**Adjusted hazard ratio (95% CI) p-value**
Respiratory disease (ref = no)	1^a^ < 0.001			
URI	3.5 (2.0, 6.2)^b^	_	_	_
Pulmonary disease	2.9 (1.7, 5.1)^b^			
ASA classification (ref = 1)	1^a^ 0.014	_	_	_
2	2.0 (0.99, 3.9)^a^			
3	2.9 (1.4, 6.1)^b^			

p-value from likelihood ratio test, ASA: American Society of Anesthesiologist; CI: Confidence interval; URI: Upper respiratory tract infection, hazard ratios within columns and variables that have no superscript (^a^^b^) in common differ significantly at p < 0.05 (Wald test).

**Table 5:** Multivariate Cox regression of laryngospasm based on period of anesthesia (n = 42,055)

**Variable**	**All periods (75)**	**Induction (27)**	**Maintenance (38)**	**Emergence (10)**
	**Adjusted hazard ratio (95% CI) p-value**	**Adjusted hazard ratio (95% CI) p-value**	**Adjusted hazard ratio (95% CI) p-value**	**Adjusted hazard ratio (95% CI) p-value**
Age (ref= > 6)		1^a^ 0.08	1^a^ 0.004	1^a^ 0.002
1-6	_	1.4 (0.57, 3.6)^a^	3.4 (1.5, 7.8)^b^	8.5 (1.8, 40.3)^b^
< 1		3.7 (1.2, 11.3)^b^	3.4 (0.99, 11.6)^a^	27.4 (5.2, 143.2)^c^
Respiratory disease (ref = no)	1^a^ 0.02		s	
URI	1.7 (0.78, 3.7)^a^	_	_	_
Pulmonary disease	3.1 (1.5, 6.4)^b^			
ASA classification (ref = 1)	1^a^ 0.23			
2	0.82 (0.47, 1.4)^a^	_	_	_
3	0.39 (0.12, 1.3)^a^			
Airway device (ref = ETT-C)	1^a^ < 0.001			
Jet ventilation-C	_			
Facemask-A	18.1 (6.4, 51.4)^b^			
LMA-A	12.5 (4.6, 33.9)^b^			
Intubating agent (ref = NDMR)		1^a^ < 0.001	1^a^ 0.67	
Succinylcholine	_	13.5 (3.9, 46.3)^b^	1.4 (0.28, 6.9)^a^	_
Sevoflurane		1.9 (0.18, 19.5)^a^	2.8 (0.6, 13.0)^a^	
Inhalation agent (ref = sevoflurane)	1^a^ < 0.001			
Isoflurane	0.75 (0.38, 1.5)^a^	_	_	_
Halothane	1.6 (0.37, 6.5)^a^			
Desflurane	11.0 (5.1, 23.9)^b^			

p-value from likelihood ratio test, ASA: American Society of Anesthesiologist; CI: Confidence interval; ETT-C: Endotracheal tube with controlled ventilation; LMA-A: Laryngeal mask airway with assisted ventilation; NDMR: Non-depolarizing muscle relaxant; URI: Upper respiratory tract infection, hazard ratios within columns and variables that have no superscript (^a^^bc^) in common differ significantly at p < 0.05.

**Table 6:** Multivariate Cox regression of upper airway obstruction in combination with reintubation based on period of anesthesia (n = 42,055)

**Variable**	**All periods (23)**	**Induction (1)**	**Maintenance (15)**	**Emergence (7)**
	**Adjusted hazard ratio (95% CI) p-value**	**Adjusted hazard ratio (95% CI) p-value**	**Adjusted hazard ratio (95% CI) p-value**	**Adjusted hazard ratio (95% CI) p-value**
Age (ref= > 6)			1^a^ 0.01	1^a^ 0.005
1-6	_	_	3.7 (0.90, 14.6)^a^	4.7 (0.90, 23.3)^a^
< 1			9.2 (2.2, 39.0)^b^	18.5 (3.7, 93.6)^b^
Site of operation (ref = superficial)	1^a^ < 0.001			
Broncho/laryngoscopy	22.8 (2.6, 201.8)^b^			
Ear-nose-throat	4.7 (0.57, 38.3)^a^	_	_	_
Eye	_			
Thoracic	0.83 (0.05, 13.3)^a^			
Abdomen	1.2 (0.13, 9.9)^a^			
Spine & extremity	1.4 (0.14, 13.7)^a^			
Remote*	_			

p-value from likelihood ratio test, CI: Confidence interval, *remote sites including radiology service and cardiac catheterization, hazard ratios within columns and variables that have no superscript (^a^^b^) in common differ significantly at p < 0.05 (Wald test).

## Discussion

The overall proportion of IRE in children undergoing GA between 2005 and 2011 in Songklanagarind Hospital was 2.23%, respectively. A longer anesthesia time (from 1 to 8.7 hr) correlated with a higher IRE probability (from 1.7% to 8%). The low incidence of IRE compared to that of other studies could be explained by the different criteria used to define desaturation in previous studies [4,8,11]. Although the proportion of IRE was highest at the maintenance period of anesthesia (43%), the incidence density per 100,000 person-minutes of any IRE was highest (61.3) at the induction period compared to the maintenance (13.7) and emergence periods (16.5).

The patient and surgery-related risk factors for IRE, namely age ≤ 6 years, having respiratory disease and undergoing airway surgery were quite similar to those reported in other studies [4-6,8]. However, history of allergic rhinitis, history of food or drug allergy and experience of key anesthesiologist were not found to be associated with IRE in our study. Previous studies did not distinguish the effects in different periods [3-8]. The factors that appeared to have different effects in each period of anesthesia in our study were age, history of respiratory disease and ASA classification for any IRE, and intubating agent for laryngospasm. Patient position and anesthesia-related risk factors for IRE i.e. airway device with mode of ventilation, using succinylcholine for intubation and using desflurane anesthesia were somewhat surprising findings best described by analysing each IRE separately as was done in this study.

Desaturation was the most common IRE (54%). A high incidence density of desaturation (44.9 per 100,000 person-minutes) was seen during the induction period of anesthesia, a result supported by Bunchungmongkol et al. [12] and Hallan et al. [13]. Light anesthesia with ETT placed during the maintenance period (31%) was the most common cause of desaturation, which might be precipitated by positioning or surgical stimulation during the first 15 minutes. Being in the lateral position increased the hazard of desaturation two-fold compared to the supine position. Laterality could affect ventilation and perfusion matching and easily lead to desaturation aggravated by light anesthesia.

The second most common cause of desaturation was laryngospasm (26%). A high rate of laryngospasm was seen in the first 30 minutes when airway stimulation might precipitate laryngospasm at the time of surgical stimulation. Assisted ventilation via facemask or LMA increased the hazard rate of laryngospasm more than 12-fold compared with controlled ventilation via ETT. However, our result contrasts with the study of Flick and Bordet et al. [14,15], in which the incidence of laryngospasm was found to be only slightly higher in children whose airway was managed with LMA (1.7%) compared to those with ETT (1.1%) and actually lower in some other studies [8,16,17]. The true explanation why our result was different compared with other studies is unknown. However, a high rate of laryngospasm at the time of surgical stimulation could possibly occur in inadequately anesthetized children whose airway was managed by spontaneous breathing with assisted ventilation via facemask or LMA rather than those receiving NMBA and controlled ventilation via ETT. Moreover, desaturation is a usual consequence of laryngospasm and could therefore be misdiagnosed as desaturation alone, especially when an ETT is in place.

Use of desflurane incurred a hazard rate of desaturation and laryngospasm 5- and 10-fold higher, respectively, compared to the use of other inhalation agents, a result supported by a study by Lerman et al. [18]. The mechanism of desflurane inducing airway contraction by activating transient receptor potential-A1 during spontaneous breathing with assisted ventilation via facemask or LMA makes laryngospasm more likely than when using airway protection with ETT [19].

Succinylcholine is used for ETT intubation in our institute in high risk children such as rapid sequence induction or difficult airway. ASA classification 3 and having respiratory disease were often considered as indicating high risk and were put into the Cox regression analysis of desaturation and laryngospasm even though they were not significant predictors. The use of succinylcholine for intubation increased the hazard rate of desaturation two-fold higher compared to use of a non-depolarizing muscle relaxant, whereas the use of succinylcholine at induction period (first 15 minutes) incurred a hazard rate of laryngospasm much higher (13.5-fold) after adjusting for ASA classification and having respiratory disease. However, the temporal relationship between succinylcholine and laryngospasm could not be determined because succinylcholine can be used as a treatment for laryngospasm [20] or for children requiring rapid sequence intubation [21]. Thus, the direction of causation should be interpreted with caution.

In three multivariate Cox regression analyses, i.e. models having IRE, desaturation and laryngospasm as the outcome, the influence of airway device was consistent. ETT protected all three adverse events. The parameter of mechanical ventilation set by standard operating procedures could not be estimated separately because the mode of ventilation was highly linked to airway device i.e. children with ETT using NMBA received controlled ventilation whereas children who breathed spontaneously through facemask or LMA received assisted ventilation. It was not possible to determine whether aiway device or mode of ventilation had a stronger effect on adverse events from our data.

Wheezing or bronchospasm (22%) was the third most common cause of desaturation in our study. A high incidence density of wheezing or bronchospasm (23.9 per 100,000 person-minutes) was seen during the induction period of anesthesia. High risk children including having URI or pulmonary disease and ASA classification 3 were the risk factors for wheezing in all periods in our study regardless of age and airway management, a finding contrary to other studies [8,16].

The incidence density of UAO in combination with reintubation was highest during the emergence period of anesthesia (3.4 per 100,000 person-minutes). UAO in combination with reintubation occurred mostly during 60-180 minutes of anesthesia when the operation was finished and the airway device was taken out. The main risk factors were age < 1 years and airway surgery, a result supported by Ing et al. [22] and Rujirojindakul et al. [23] Anesthesia-related risk factors could not be determined because of the low number (23) of events.

### Strengths and limitations

The strength of this study was its use of time-to-event analysis, which takes into account the changing risk of IRE over the duration of GA and incidence density per person-time, which has not been done in previous studies in children. Because of using routinely recorded data, a limitation was that some patient-related risk factors, such as history of parental smoking or passive smoking and obstructive sleep apnea syndrome, were not available in the assessment. Although the historical cohort design may result in information bias from under-reporting, which can underestimate the true incidence [24], we managed to determine statistically significant surgery- and anesthesia-related risk factors including those with time-varying effects. Moreover, the accuracy of the study in terms of internal validity was quite high due to the absence of false positives, the low rate of missing data (1.4%) and the large sample size. Because of the high internal validity, the generalizability to other child populations should be persuasive even though a single hospital was used to recruit subjects [25].

## Conclusions

The risk of most forms of IRE except UAO and of any IRE was highest during the induction and early maintenance periods of anesthesia (first 30 minutes of anesthesia). The common risk factors for IRE at any time were airway surgery, desflurane anesthesia and airway device with mode of ventilation. The effect-varying risk factors of any IRE were age, history of pulmonary disease and ASA classification. Therefore, anesthesiologists should pay more attention during the induction and early maintenance periods especially when certain airway devices incorporating assisted ventilation or desflurane are used.

## Abbreviations

IRE: Intraoperative respiratory events; PRE: Perioperative respiratory events; URI: Upper respiratory tract infection; GA: General anesthesia; ASA: American Society of Anesthesiologist; ETT: Endotracheal tube; NMBA: Neuromuscular blocking agent; PACU: Post-anesthetic care unit; LMA: Laryngeal mask airway; PICU: Pediatric intensive care unit; UAO: Upper airway obstruction.

## Competing interests

The authors declare that they have no competing interest.

## Authors’ contributions

All those listed as authors contributed to the preparation of the manuscript. Each listed author participated in the work that they can defend its content. MO coordinated the study, participated in the study design, undertook the statistical analysis and wrote the draft manuscript. AFG participated in the study design, undertook the statistical analysis and revised the draft manuscript. VC participated in the study design and revised the draft manuscript. NP participated in the study design and wrote the draft manuscript. KN participated in the study design and coordinated the drafting of the manuscript. All authors read and approved the final version.

## Pre-publication history

The pre-publication history for this paper can be accessed here:

http://www.biomedcentral.com/1471-2253/14/13/prepub

## Supplementary Material

Additional file 1 Univariate Kaplan Meier analysis of intraoperative respiratory events (n = 14,153). Click here for file
